# Glutamine Depletion Induced Senescence-Associated β-Galactosidase Activity and Impaired Functional Properties of Ea.hy926 Endothelial Cells

**DOI:** 10.3390/cells15121116

**Published:** 2026-06-20

**Authors:** Jamil Haider, Xiaoyan Huang, Chunyu Xu, TinChung Leung

**Affiliations:** 1 North Carolina Research Campus, The Julius L. Chambers Biomedical/Biotechnology Research Institute, North Carolina Central University, Kannapolis, NC 28081, USA; 2 Department of Biological & Biomedical Sciences, North Carolina Central University, Durham, NC 27707, USA

**Keywords:** glutamine, senescence-associated beta-galactosidase activity, Ea.hy926 endothelial cell, zebrafish

## Abstract

Glutamine is a conditionally essential amino acid that is important for endothelial homeostasis, while endothelial cell dysfunction is associated with altered glutamine metabolism and shifts toward stress-responsive pathways. We investigated the role of glutamine and senescence-associated beta-galactosidase (SA-β-gal) activity in Ea.hy926 endothelial cells (ECs), together with supportive functional activity assays. We found that glutamine depletion induced a progressive decline in endothelial function. Specifically, glutamine-depleted ECs exhibited increased SA-β-gal activity, accompanied by impaired proliferative capacity, disrupted cellular morphogenesis, increased promyelocytic cell adhesion, and diminished ability to promote host tissue proliferation and EC morphogenesis in a zebrafish xenograft model. These findings suggest that glutamine availability is crucial for maintaining endothelial integrity and functional competence.

## 1. Introduction

Glutamine is a conditionally essential amino acid that serves as a fundamental metabolic substrate for nearly all mammalian cells [1]. It functions as a primary carbon and nitrogen donor for biosynthetic processes and energy production, contributing to redox balance, nucleotide synthesis, and cellular homeostasis. In cell culture systems, glutamine is often supplemented at higher concentrations than other amino acids to meet the substantial energetic and biosynthetic demands of proliferating cells [2]. Beyond its metabolic role, glutamine is critical for the functioning of the immune and gastrointestinal systems and is required for the synthesis of proteins, nucleic acids, and other macromolecules.

Importantly, glutamine has been identified as a key regulator of the Akt-mTOR signaling axis, a pathway that integrates intracellular and extracellular cues to modulate growth, proliferation, and survival [3,4,5]. Chronic glutamine depletion has been shown to activate mTOR signaling while impairing autolysosome function, a combination that disrupts autophagy and ultimately driving EC dysfunction. Conversely, glutamine repletion can attenuate mTOR activation and restore autophagic flux, alleviating EC dysfunction-associated impairment [6].

Increased SA-β-gal activity is a hallmark of vascular pathology and a major contributor to cardiovascular disease. In ECs, this state is characterized by a metabolic decline and dysregulation of endothelial-derived mediators such as nitric oxide, endothelin-1, prostaglandins, thromboxane, vascular endothelial growth factor and angiotensin-converting enzyme. These molecules regulate vascular tone, coagulation, inflammation, oxidative stress, immune cell trafficking, and metabolic homeostasis [7]. Endothelial cells play an important role in the pathogenesis of cardiovascular diseases, including atherosclerosis, which is a chronic inflammatory condition marked by endothelial injury, lipid accumulation, and arterial stiffening [8]. The endothelium, composed of a monolayer of ECs lining the vasculature, plays a pivotal role in maintaining vascular integrity. Persistent EC dysfunction, combined with oxidative stress and chronic inflammation, is strongly linked to impaired vascular homeostasis and elevated cardiovascular risk [9,10].

Evaluating SA-β-gal activity in ECs may provide useful information to identify strategies for mitigating vascular disease. Several non-essential amino acids, including glutamine, become conditionally essential under conditions of metabolic stress, injury, or endothelial dysfunction. Amino acids such as arginine, cysteine, glycine, proline, tyrosine, and taurine, alongside glutamine, have been implicated in the regulation of vascular health and the development of atherosclerosis [11,12]. Glutamine, in particular, has shown antioxidant, anti-inflammatory, and anti-apoptotic properties in cardiovascular contexts [13]. Additionally, it may promote vascular health by serving as a precursor for arginine and subsequently nitric oxide synthesis, while also improving metabolic risk factors such as dyslipidemia, insulin resistance, hypertension, and obesity [14,15,16,17].

One key metabolic shift associated with increased SA-β-gal activity in ECs is the alteration in glutamine utilization. In healthy ECs, glutamine is predominantly catabolized via glutaminolysis, feeding the tricarboxylic acid (TCA) cycle to support energy production [18]. However, when ECs do not function properly, glutamine metabolism is redirected toward biosynthetic pathways, such as the hexosamine biosynthetic pathway, which mediates the glycosylation of nucleotides, proteins, and lipids [19]. This metabolic reprogramming contributes to increased oxidative stress and inflammatory signaling, and plays a role in cardiac pathophysiology [20]. Dysregulation of this pathway may further exacerbate endothelial dysfunction. Importantly, SA-β-gal activity is not the sole driver of dysfunction but acts in concert with oxidative stress, inflammation, and impaired metabolic flexibility to accelerate vascular pathology. Indeed, glutamine metabolism is increasingly recognized as a key regulator of angiogenesis, vascular remodeling, endothelial dysfunction, and atherogenesis [21].

In the present study, we evaluated the role of glutamine availability in SA-β-gal activity using the human-derived Ea.hy926 ECs. Our in vitro experiments demonstrated that glutamine was essential for maintaining healthy ECs. Short-term glutamine depletion resulted in pronounced functional impairment in ECs, including a dose- and time-dependent increase in SA-β-gal activity. Additional functional assays including cell proliferation, Matrigel morphogenesis, and adhesion analyses further corroborated our findings based on SA-β-gal activity. Moreover, glutamine supplementation at the recommended concentration (2.0 mM) promoted the formation of cell mass-like structures in xenografted zebrafish embryos.

## 2. Materials and Methods

### 2.1. Cell Culture

The Ea.hy926 human endothelial cell line was originally generated and kindly provided by Dr. Cora-Jean S. Edgell at the University of North Carolina at Chapel Hill [22]. L-glutamine (referred to as glutamine), a non-essential amino acid, is unstable. In liquid media, glutamine undergoes spontaneous non-enzymatic decomposition, generating ammonia and pyroglutamate as degradation byproducts. To avoid this, we used Dulbecco’s Modified Eagle Medium (DMEM) in powder form (Corning 90-013-PB, Corning, NY, USA) without L-glutamine and added Glutamax (Gibco 35050-061, Miami, FL, USA) as stable L-glutamine equivalents separately (as per requirement). Therefore, 0.0 mM or 2.0 mM glutamine refers to L-glutamine equivalents added to DMEM throughout the manuscript. Since FBS typically contains an average L-glutamine concentration ranging between 0.5 and 1.0 mM, supplementation of DMEM with 10% FBS would contribute approximately 0.05–0.1 mM residual glutamine to the culture medium. This residual glutamine was present across all experimental conditions, including media supplemented with 2.0 mM, 1.0 mM, 0.3 mM, and 0.0 mM L-glutamine equivalents (GlutaMAX, Gibco, Miami, FL, USA). Prepared media, along with 10% fetal bovine serum (FBS) (Gemini Bioproducts 900-108, West Sacramento, CA, USA) and 2% Penicillin-Streptomycin (MP Biomedicals 1670049, Irvine, CA, USA), was used for EC culture. Although mycoplasma testing was not performed, the Ea.hy926 ECs used in this study did not show any signs suggestive of mycoplasma contamination. Cells maintained under the 2 mM glutamine control condition exhibited normal proliferation and viability. Furthermore, Hoechst and DAPI staining performed during the cell proliferation assays did not reveal detectable extranuclear, filamentous, or particulate DNA structures on the cell surface that might indicate mycoplasma contamination.

### 2.2. SA-β-Gal Assay

Dissociated (Accutase, Invitrogen, Waltham, MA, USA) cells were counted and 6 × 10^5^ cells were plated in each 60 mm cell culture treated plates. Cells were grown overnight in their respective media for reattachment. The next day, cells were washed with phosphate-buffered saline (PBS) (pH 7.0) and fixed with 4% paraformaldehyde for 5 min at room temperature. Fixed cells were washed with PBS (pH 7.0 and subsequently pH 6.0) again and 2 mL of filtered staining solution was added to each plate. 1× SA-β-gal staining solution contained 40 mM citric acid/sodium phosphate solution (pH 6.0), 150 mM sodium chloride, 2 mM magnesium chloride, 1 mg/mL 5-bromo-4-chloro-beta-D-galactopyranoside solution, 5 mM potassium ferricyanide and 5 mM potassium ferrocyanide in Millipore deionized water. Cells in staining solution were incubated at 37 °C for 6–7 h and monitored for blue color development. Reaction was stopped at the end by using deionized water replacing the staining solution. Cells were kept in deionized water for imaging. Stained cells were imaged under a Nikon dissecting microscope (SMZ1500) with a Nikon camera (DS-Fi3) (Nikon, Melville, NY, USA). Software NIS-Elements D 5.30.01 64 bit was used for imaging. All images within each experiment were captured using identical exposure settings and analyzed in ImageJ (version 1.52e) using the same threshold settings across all experimental groups. As described in the figure legends, a consistent threshold range (0–110) was applied to identify and quantify SA-β-gal-positive cells (highlighted in red).

### 2.3. EC Proliferation Assay

A total of 10,000 cells/well were pre-seeded overnight with ECs from 2.0 mM or 0.0 mM glutamine cultures into 96-well plates. The next day (Day 0), the medium was changed to 2.0 mM glutamine in DMEM complete medium at the start of the proliferation assay. Starting from Day 0, then Day 2 and Day 4, samples of ECs from the 96-well plates were detached with Accutase (Invitrogen, Waltham, MA, USA) and collected for DAPI (Invitrogen, Waltham, MA, USA)/Hoechst (Invitrogen, Waltham, MA, USA) staining. Stained cells were suspended in PBS, transferred into black-walled 96-well plates, and then subjected to cell counting using ImageXpress Pico (Molecular Devices, San Jose, CA, USA). Plates were centrifuged at 500 rpm (50× *g*) for 2 min to bring suspended cells to the bottom. The “Cell Counting” application of ImageXpress with bright-field and DAPI fluorescent setting were selected. Once inside the ImageXpress chamber, plates were subjected to cell counting of DAPI/Hoechst fluorescently labeled ECs within the selected range of cell sizes according to manufacturer instructions. The counted cells were analyzed and a graph was plotted for total number of ECs in each group.

### 2.4. Matrigel Morphogenesis Assay

A 24-well plate and Matrigel (BD#354234, BD Biosciences, San Jose, CA, USA) aliquots were kept at 4 °C on ice for 48 h before plating. A day before, 150 µL Matrigel was plated in each well using cold pipette tips. Dissociated (Accutase) cells were counted and 3 × 10^5^ cells (in 200 µL) were plated in each well of the plate on top of the Matrigel. The next day, tube-like structures were formed, which were imaged under a Nikon dissecting microscope with a Nikon camera and NIS-Elements D 5.30.01 64 bit software (Nikon, Melville, NY, USA).

### 2.5. EC-HL-60 Cell Adhesion Assay

EC was initially cultured for two weeks in DMEM supplemented with 2.0 mM glutamine. Subsequently, one group of ECs was maintained in 2.0 mM glutamine for an additional month, and considered as control. A second group was cultured in glutamine-free medium (0.0 mM glutamine) for an additional month.

For co-culture experiments with the human promyelocytic cell line HL-60, comparable numbers of control ECs and glutamine-depleted ECs were seeded into 96-well plate a day earlier before the addition of HL-60 cells. ECs were allowed to attach overnight before co-culture. Cell seeding densities for each treatment group were determined in preliminary experiments to ensure that approximately 4 × 10^4^–4.5 × 10^4^ ECs remained per well after washing. Each treatment condition was performed with six biological replicates. The next day, well-grown HL-60 cells in Iscove’s Modified Dulbecco’s Medium (IMDM, Gibco, Miami, FL, USA) (20% FBS) were labeled with Vybrant Chloromethyl-1,1′-Dioctadecyl-3,3,3′,3′-tetramethyl indocarbocyanine iodide (CM-DiI) (Invitrogen, Waltham, MA, USA) (dsRed fluorescence) dye (5 µM in media without FBS). The labeling was performed in an incubator with 5% carbon dioxide for 1 h. After washing the dye from the cells, 1.5 × 10^4^ of the stained cells were added to each 96-well plate containing ECs. After 3 h of co-culture, cells were washed twice carefully by pipetting slowly using 400 µL media. Stained HL-60 cells were counted with an image cytometer (ImageXpress Pico, Molecular Devices, San Jose, CA, USA).

### 2.6. EC Transplantation into Zebrafish Embryo

The zebrafish (*Danio rerio*) colony was maintained in zRack Stand-alone Systems (Aquaneering, San Marcos, CA, USA) with a 14 h light and 10 h dark cycle. Zebrafish AB wildtype and transgenic lines *Tg(fli1:egfp)^y1^* have been described previously [23]. Identified *Tg(fli1:egfp)^y1^* adult fish carriers of the heterozygous cross AB wildtype were used and embryos were staged and maintained according to standard protocols [24,25]. All experimental protocols and procedures are approved by the Animal Care and Use Committee of North Carolina Central University (Durham, NC) (North Carolina Central University, Institutional Animal Care and Use Committee Protocol # TCL-07-14-2008). Zebrafish embryos were euthanized to alleviate suffering by 4 mg/mL tricane (MS222) according to North Carolina Central University, Institutional Animal Care and Use Committee guidelines. The developmental stage of zebrafish embryos was staged as hour-post-fertilization (hpf) or day-post-fertilization (dpf) and grown under standard condition of 28.5 °C with 10 h/14 h day/night cycle. Zebrafish eggs were incubated at 28.5 °C in 0.3× Danieau’s solution (19.3 mM sodium chloride, 0.23 mM potassium chloride, 0.13 mM magnesium sulfate, 0.2 mM calcium nitrate, 1.7 mM 4-(2-hydroxyethyl)-1-piperazineethanesulfonic acid, pH 7.0), at 3 hpf, and eggs were aligned on an agar plate for microinjection. Cells were labeled with 5 µM of CM-DiI cell tracker dye a day before the grafting procedure. The injection volume and cell suspension was calibrated to 200–300 cells/injection in each egg. After transplantation, eggs were kept in 0.3× Danieau’s solution until 8 hpf and subsequently changed to 0.3× Danieau’s solution containing 1× 1-phenyl-2 thiourea (30 µg/mL) to inhibit the development of pigmentation. At 24 hpf, zebrafish embryos were dechorionated using 150 mg/mL of trypsin (Sigma, St. Louis, MO, USA) for 1 h and embryos were kept in 0.003% phenylthiourea for further use. At 6 dpf, zebrafish embryos were analyzed with Olympus MVX10 macroscope (Olympus, Center Valley, PA, USA), connected with vertebrate automated screening technology VAST BioImager (Union Biometrica, Holliston, MA, USA).

### 2.7. Statistical Analysis

One-way ANOVA was used to determine whether significant differences existed among three or more independent groups. When the ANOVA indicated a significant effect, Student’s *t*-test was subsequently performed to identify specific pairwise differences between groups. Data are presented as the mean ± standard error of the mean (SEM) of biological replicates for each experiment. The results were confirmed by repeats of two independent experiments. The observed differences were regarded as significant if the calculated two-tailed probability (*p*) values * *p* < 0.05, ** *p* < 0.01 and *** *p* < 0.001.

## 3. Results

### 3.1. SA-β-Gal Activity Was Enhanced in Glutamine-Depleted ECs

**Figure 1:**
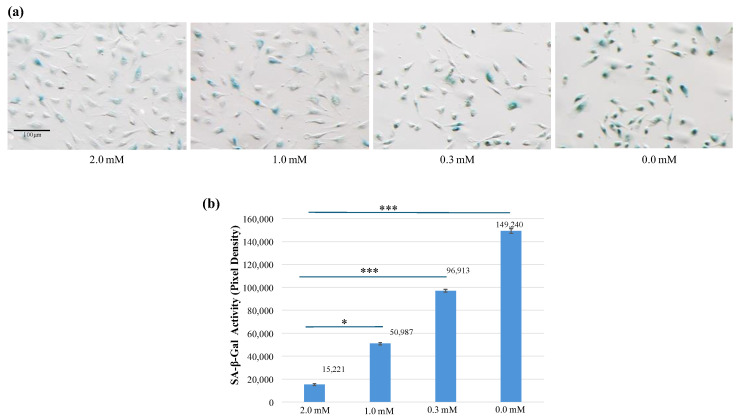
SA-β-gal activity increased in a dose-dependent manner with glutamine depletion. (**a**) SA-β-gal activity was evaluated after 13 days of exposure to four glutamine concentrations (2.0, 1.0, 0.3, and 0.0 mM), with 2.0 mM serving as the control condition. (**b**) SA-β-gal activity was quantified as pixel density using ImageJ software. Quantitative analysis demonstrated a progressive increase in SA-β-gal activity with decreasing glutamine concentrations. One-way ANOVA followed by Student’s *t*-test revealed significant (* *p* < 0.05) to highly significant (*** *p* < 0.001) increases in SA-β-gal activity associated with glutamine depletion. Groups: 2.0 mM, 1.0 mM, 0.3 mM, and 0.0 mM; independent experimental replicates N = 3 per group. Scale bar = 100 µm.

**Figure 2:**
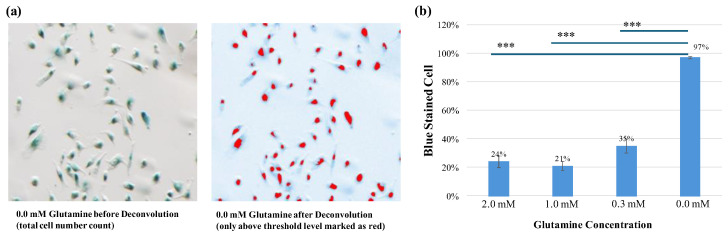
Quantification of SA-β-gal-positive cells increased under glutamine depletion. Following image color deconvolution in ImageJ and selection of the “Alcian blue” channel, a consistent threshold range (0–110) was applied to identify and quantify SA-β-gal-positive cells (highlighted in red). (**a**) Representative image illustrating cell detection and thresholding parameters used for quantification. (**b**) The proportion of SA-β-gal-positive cells was determined relative to total cell counts. Quantitative analysis demonstrated a dose-dependent increase in SA-β-gal activity with decreasing glutamine concentrations. One-way ANOVA followed by Student’s *t*-test indicated a highly significant increase (*** *p* < 0.001) in SA-β-gal activity under glutamine-depleted conditions. Groups: 2.0 mM, 1.0 mM, 0.3 mM, and 0.0 mM; independent experimental replicates N = 3 per group. Scale bar = 100 µm.

**Figure 3:**
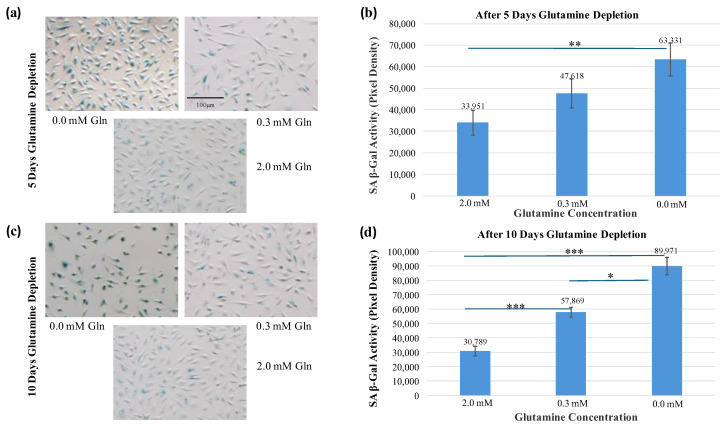
Endothelial SA-β-gal activity increased with prolonged glutamine depletion. (**a**,**c**) SA-β-gal staining intensity was greater after 10 days of glutamine depletion compared with 5 days. (**b**,**d**) Quantitative analysis of SA-β-gal activity following 5- and 10-day glutamine depletion. One-way ANOVA followed by Student’s *t*-test showed an approximately three-fold increase in SA-β-gal activity after 10 days under 0.0 mM glutamine conditions compared to control (2.0 mM glutamine), which was highly significant (*** *p* < 0.001). At 5 days, the increase was less than two-fold but remained significant (** *p* < 0.01). Groups: 2.0 mM, 0.3 mM, and 0.0 mM; independent experimental replicates N = 3 per group. * *p* < 0.05. Scale bar = 100 µm.

**Figure 4:**
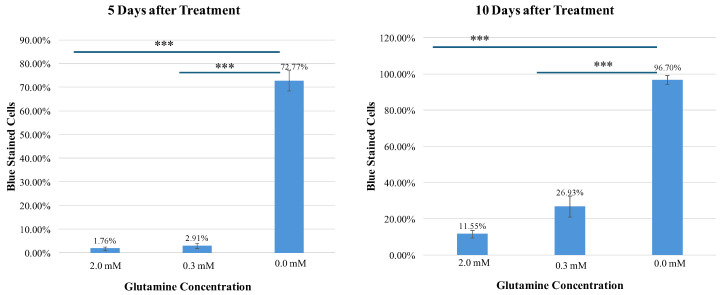
Quantitative analysis confirmed time- and dose-dependent effects of glutamine depletion on percentage of SA-β-gal-positive cells. Image deconvolution and threshold-based (range 0–110) quantification of SA-β-gal-stained cells demonstrated patterns consistent with those shown in Figure 3, with elevated SA-β-gal activity under reduced glutamine conditions. Quantification of the proportion of SA-β-gal-positive cells across three glutamine concentrations revealed a significant increase with decreasing glutamine availability. One-way ANOVA followed by Student’s *t*-test indicated that at 5 days, cultures maintained in 0.0 mM glutamine exhibited a highly significant increase (*** *p* < 0.001), with ≥70% of cells positive for SA-β-gal relative to the 2.0 mM control. This effect was further pronounced after 10 days, with SA-β-gal-positive cells approaching 90% (*** *p* < 0.001). Groups: 2.0 mM, 0.3 mM, and 0.0 mM; independent experimental replicates N = 3 per group.

SA-β-gal activity is a widely used and robust biomarker detected at suboptimal lysosomal pH (6.0). This enzymatic activity reflects the heightened lysosomal mass and altered metabolic state. Firstly, we demonstrated a dose-dependent effect of glutamine depletion over a 13-day period (Figure 1 and Figure 2), where Figure 1 shows the SA-β-gal activity in blue color intensity and Figure 2 shows the percentage of SA-β-gal-positive (blue) cells out of the total. Secondly, we demonstrated the time-dependent effect of glutamine depletion at the 5-day and 10-day time points (Figure 3 and Figure 4), where Figure 3 shows the activity in blue color intensity and Figure 4 shows the percentage of SA-β-gal-positive (blue) cells.

The Ea.hy926 ECs were routinely cultured in DMEM supplemented with 2.0 mM glutamine. Based on our microscopic observations of impaired proliferation under glutamine depletion, we performed a SA-β-gal assay, a widely used method for assessing cellular dysfunction, in ECs treated with varying glutamine concentrations (0.0, 0.3, 1.0, and 2.0 mM) for 13 days. The 13-day time point was selected based on our observation that glutamine-depleted EC ceased proliferation and some cells died and washed away at the first passage, and the rest of the cells entered a quiescent, non-proliferative state. Despite this growth arrest, the remaining cells were viable in culture, retaining the ability to reattach to culture plates upon replating over 2–3 subsequent passages, corresponding to 13 days, relative to control ECs. The use of multiple glutamine concentrations allowed us to evaluate potential dose-dependent effects on ECs. As expected, we observed a dose-dependent increase in the proportion of ECs exhibiting morphological changes, including cellular flattening and enlargement, accompanied by enlarged and frequently irregularly shaped nuclei. The highest glutamine concentration (2.0 mM), representing the standard culture condition, served as the control (Figure 1).

SA-β-gal assay showed a clear increase in intensity and number of stained cells with increasing glutamine depletion (Figure 1a). Intensity of bluish-green cells, measured by ImageJ showed that ECs without glutamine supplement exhibited the most intense coloration followed gradually by 0.3 mM and 1.0 mM glutamine groups. Control ECs (2.0 mM) showed the least intensity. Endothelial cell cultures maintained under glutamine-free condition (0.0 mM glutamine) exhibited an approximately 10-fold increase in SA-β-gal staining compared to control cultures supplemented with 2.0 mM glutamine. Similarly, intermediate concentrations of glutamine, 0.3 mM and 1.0 mM, indicated 6.4- and 3.4-fold increase compared to the control (Figure 1b), respectively, which were statistically significant at *** *p* < 0.001 and * *p* < 0.05 level. This provided strong evidence that increased SA-β-gal activity was dose-dependent upon glutamine depletion. In addition to the measurement of intensity of bluish-green cells using ImageJ (Figure 1), we quantified the percentage of SA-β-gal-positive cells across treatment groups (Figure 2). Quantification of the percentage of SA-β-gal-positive cells also revealed increased activity under glutamine-depleted conditions (0.0 mM glutamine), although this method was less sensitive than measurement of staining intensity.

In our subsequent experiment, we sought to determine the temporal onset and magnitude of alterations induced by glutamine depletion in ECs. Endothelial cells were assessed after 5- and 10-day glutamine depletion, rather than 13 days, using the same glutamine concentrations as in the previous assay (Figure 3). Figure 3a indicated an increase in SA-β-gal activity as early as 5 days under glutamine depletion. Those cells cultured for 5 days in 0.3 mM and 0.0 mM glutamine had an almost 1.4- and 1.9-fold increase in SA-β-gal activity compared to the control culture at 2.0 mM glutamine and was statistically highly significant (Figure 3a,b). The 10-day glutamine-depleted cells in the same figure also showed the same trend as the 5-day treatment. At this time point, ECs were more vulnerable than at the earlier 5-day stage, exhibiting a 1.9-fold and 2.9-fold increase in SA-β-gal intensity in the 0.3 mM and 0.0 mM glutamine conditions, respectively, compared to the 2.0 mM glutamine control (Figure 3c,d). This effect was highly statistically significant (*** *p* < 0.001). We detected a dose-dependent response of glutamine depletion on SA-β-gal activity. Even the intermediate glutamine concentration (0.3 mM) indicated a highly significant difference. In addition, we quantified the percentage of SA-β-gal-positive cells across both treatment groups in 5-day and 10-day glutamine depletion (Figure 4). Both quantitative methods gave similar conclusions that glutamine depletion increased SA-β-gal activity in ECs in a dose-dependent manner in as soon as 5- and 10-day treatments. This provided evidence that glutamine depletion dysregulated SA-β-gal activity in Ea.hy926 ECs.

Glutamine depletion induced a robust, dose- and time-dependent increase in SA-β-gal activity in Ea.hy926 ECs, detectable as early as 5 days and markedly elevated by 10 and 13 days. Reduced glutamine availability (0.0–1.0 mM) significantly increased both SA-β-gal staining intensity and the proportion of SA-β-gal-positive cells compared to control conditions (2.0 mM), concomitant with growth arrest and morphological changes. These findings demonstrated that glutamine depletion rapidly disrupted Ea.hy926 EC homeostasis and promoted lysosome-associated SA-β-gal activity, a recognized hallmark of endothelial dysfunction.

### 3.2. Glutamine Depletion in ECs Resulted in Diminished Proliferation Activity

**Figure 5:**
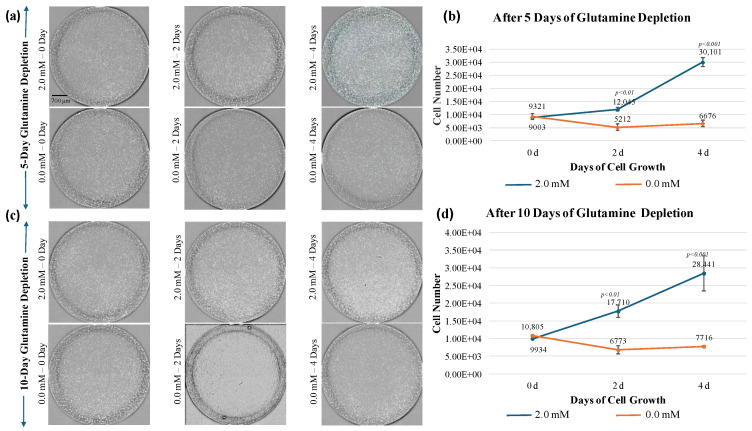
Endothelial cell proliferation was markedly reduced under glutamine-depleted conditions. (**a**,**c**) Representative images of EC in 96-well plates used for quantification of total cell number following DAPI and Hoechst staining. (**b**,**d**) Graphical representation of EC counts derived from panels (**a**,**c**). Cell proliferation was substantially reduced in cultures maintained without glutamine at both time points (5 and 10 days), with minimal increases or gradual declines in cell number. In contrast, control ECs maintained at 2.0 mM glutamine displayed consistent proliferative expansion over time. One-way ANOVA followed by Student’s *t*-test was performed between glutamine conditions within the same time point. Groups: 2.0 mM and 0.0 mM; independent experimental replicates N = 4 per group. Scale bar = 700 µm.

Angiogenic ECs have high rates of glycolysis and glutaminolysis, and both pathways can provide mostly needed biosynthetic intermediates and energy for sprouting and cell proliferation [26]. Therefore, it prompted us to investigate the effect of glutamine depletion on EC proliferation (Figure 5). Glutamine is an important amino acid supplement commonly added to mammalian cell culture media and serves as an auxiliary energy source, especially when cells are rapidly dividing. Culturing the human Ea.hy926 ECs under a glutamine-depleted condition for 5 and 10 days resulted in a pronounced reduction in cell number (Figure 5a,b). Same numbers of control (2.0 mM glutamine) and glutamine-depleted cells (0.0 mM glutamine) were plated in 96-well plates and incubated overnight at 37 °C with 5% carbon dioxide before counting at 2 and 4 days with an image cytometer (ImageXpress Pico, Molecular Devices). During subculturing of the ECs, glutamine-depleted cells exhibited reduced proliferative capacity, whereas detached or non-viable cells were routinely removed during bi-weekly media changes. While a certain degree of cell death is a common occurrence during culture, it was notably elevated under glutamine-depletion conditions. It is important to note that these ECs subjected to assays were adherent and viable; notably, glutamine-depleted cells retained the ability to reattach to culture plates following twice-weekly replating prior to the proliferation assays. Results indicated (Figure 5c,d) a 1.3- and 3.2-fold increase in cell number with control ECs (2.0 mM glutamine) for 2- and 4-day proliferation assays, respectively. In contrast, cells subjected to glutamine depletion for 5 days failed to proliferate; instead, cell numbers were reduced to 56% and 72% compared to initial Day 0 for 2- and 4-day cell proliferation assays, respectively. A similar trend was found also in 10-day glutamine-depleted cells. Control cells (2.0 mM glutamine) had a 1.7- and 2.6-fold increase for 2- and 4-day cell proliferation assays, respectively, whereas glutamine-depleted cells showed a decrease to 63% and 71% compared to initial Day 0 for 2- and 4-day cell proliferation assays, respectively. The cell proliferation assays showed statistically significant differences in 2-day (** *p* < 0.01) and 4-day (*** *p* < 0.001) groups (Figure 5d).

### 3.3. Endothelial Cells with Glutamine Depletion Exhibited Defective Vascular Morphogenesis

**Figure 6:**
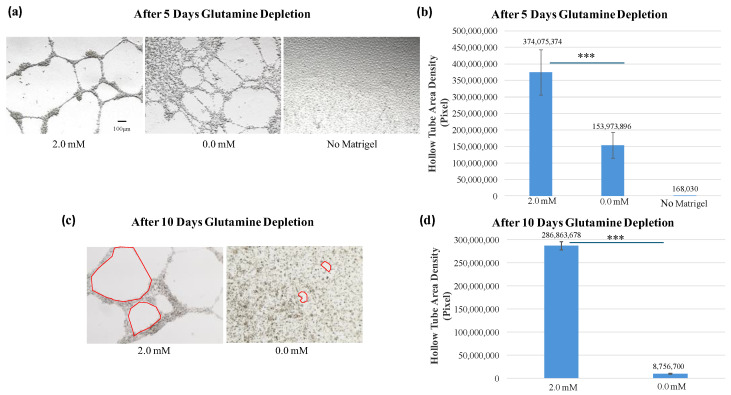
EC tube-like structure formation was impaired by glutamine depletion. (**a**,**c**) Endothelial tube-like structure formation assays performed after 5-day and 10-day exposure to glutamine depletion. Control endothelial cells (2.0 mM glutamine) formed characteristic hollow tube-like networks, whereas glutamine-depleted cultures displayed a reduced capacity to form organized structures. (**b**,**d**) Quantification of tube-like structure area from panels (**a**,**c**). After 5 days of glutamine depletion, cells maintained at 0.0 mM glutamine showed a reduction to approximately 41% of the hollow tube-like area observed in control cultures (**b**). Cells plated without a Matrigel matrix did not form tube-like structures (**a**). Following 10 days of glutamine depletion, ECs largely lost tube-like structure formation capacity (**d**). Red-outlined regions illustrate representative hollow tube-like areas quantified by pixel density using ImageJ (**c**). One-way ANOVA followed by Student’s *t*-test was performed, (*** *p* < 0.001). Five plates were analyzed for each glutamine condition, except the no-Matrigel group (three plates). For the 5-day assay: 2.0 mM (independent experimental replicates N = 5), 0.0 mM (N = 5), and no Matrigel (N = 3). For the 10-day assay: 2.0 mM (N = 5) and 0.0 mM (N = 5). Scale bar = 100 µm.

**Figure 7:**
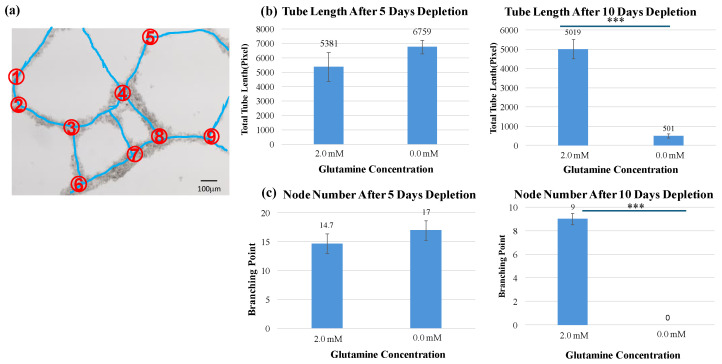
Prolonged glutamine depletion resulted in loss of tube-like structure formation. (**a**) Schematic representation of tube-like structure network quantification illustrating measurements of total tube length (blue lines) and branching nodes (red numbers). Tube length and node number were quantified using ImageJ. (**b**,**c**) After 5 days of treatment, no significant differences were observed in total tube length or node number between control (2.0 mM glutamine) and glutamine-depleted (0.0 mM) conditions. However, after 10 days of glutamine depletion, endothelial cells exhibited a near-complete loss of tube-like-forming capacity, with minimal node formation and a highly significant reduction in total tube length (*** *p* < 0.001). For both 5-day and 10-day assays: 2.0 mM (independent experimental replicates N = 3) and 0.0 mM (N = 3). Scale bar = 100 µm.

Glutamine is the most abundant circulating amino acid and a critical metabolic substrate for ECs, supporting protein synthesis, TCA cycle anaplerosis, redox balance, and proliferative capacity [27,28]. While some studies report that glutamine metabolism is dispensable for short-term EC migration [28], others suggest it is essential for tip-stalk cell dynamics and migratory behavior [27], creating an unresolved discrepancy in the field. To address this, we investigated how glutamine depletion would influence EC morphogenesis using a Matrigel-based tube-like structure formation assay, a well-established in vitro model of angiogenesis in which ECs form capillary-like networks in response to extracellular matrix cues and angiogenic factors [29,30,31]. By evaluating EC tube-like structure-forming capacity after 5- and 10-day glutamine depletion (Figure 6 and Figure 7), this study aimed to provide new insights into the role of glutamine in regulating endothelial morphogenesis and to clarify its contribution to EC morphogenesis behavior.

We assessed the EC morphogenesis ability by two methods. Firstly, we measured tube-like structure area (hollow tube-like area) density using ImageJ. Initially we observed a partial tube-like formation in 5-day glutamine-depleted EC. However, for the 5-day treatment group, the control ECs (2.0 mM glutamine) exhibited extensive hollow tube-like areas in the entire well, which was an indicator of Matrigel morphogenesis in ECs. As a negative control, ECs were cultured in the absence of extracellular matrix to confirm that tube-like structure formation did not occur without matrix support (Figure 6a). Five-day glutamine-depleted cells resulted in a decrease to 41% compared to the control (2.0 mM glutamine) in tube-like formation process, which was statistically significant (*** *p* < 0.001) (Figure 6b). When ECs were cultured with glutamine depletion for an extended time period (10 days), the tube-like structure was almost completely lost in glutamine-depleted (0.0 mM glutamine) cells (Figure 6c). These cells exhibited a reduction in tube-like structure forming area to 3% compared to the control (2.0 mM glutamine), a negligible response indicating an inability to mount a morphogenesis response to Matrigel (Figure 6d). This study specifically aimed to measure the capacity of Ea.hy926 EC to form tube-like structures, a fundamental characteristic of angiogenesis. In this assay, ECs were cultured as a nearly confluent monolayer, and lumen-like formation was induced using Matrigel. This method contrasted with other EC assays in which cells were seeded at low densities, preventing confluency and resulting in the formation of interconnected networks resembling a “fishnet” pattern. These networks were defined by branching points and elongated cell–cell aggregations that mimic tubular connections but lack lumen-like structures. In this study, we provided a distinct assay for evaluating EC lumen-like formation, and lumen-like area was quantitatively assessed (Figure 6). Secondly, we also measured different EC morphogenesis parameters in the same experiment to quantify total tube-like length and the number of branching points as the nodes, and incorporated these analyses into Figure 7. Similarly, 10-day glutamine depletion almost abolished the ability of ECs to undergo Matrigel morphogenesis. Taken together, these ECs (0.0 mM glutamine) exhibited elevated SA-β-gal activity, lost cell proliferation activity and defective vascular morphogenesis. They all pointed toward dysfunctional EC phenotypes. These observations prompted us to further examine the effect of glutamine depletion on promyelocytic cell–EC adhesion as well as EC interaction with zebrafish host cells in a xenograft model.

### 3.4. Glutamine Depletion Promoted Promyelocytic–Endothelial Cell Adhesion

Glutamine is a major regulator of immune function, influencing both myeloid and lymphoid cells [32,33]. Immune cells such as lymphocytes, neutrophils and macrophages consume glutamine at high rates [34]. Because of this, glutamine might be considered as a fuel for the immune system and its availability in blood circulation might regulate immune function and impact clinical outcomes [1,35]. Glutamine depletion was implicated with immune and vascular cell dysfunction in Coronavirus Disease of 2019 (COVID-19) patients and associated with risk of lethal pulmonary and vascular complications [36], and glutamine depletion was an independent predictor of hospital mortality in intensive care unit patients [35]. It was supported by other studies on intensive care unit patients who needed parenteral nutrition (intravenously) that glutamine supplementation had shown reduced mortality [37,38].

**Figure 8:**
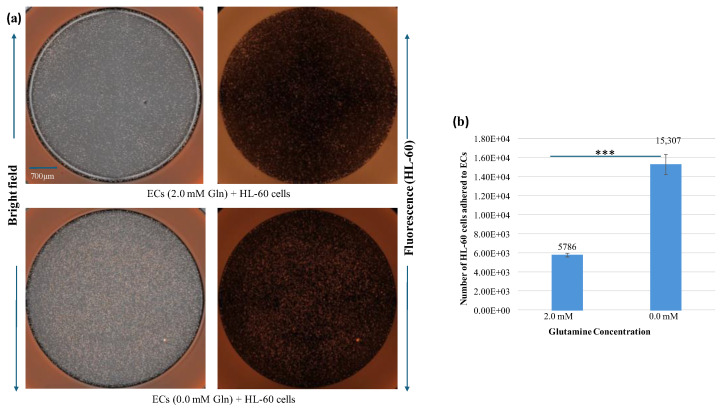
Glutamine-depleted ECs exhibited increased HL-60 promyelocytic cell adhesion. The promyelocytic cell line HL-60 was incubated with equivalent numbers of ECs maintained under different glutamine conditions. (**a**) Representative bright-field and fluorescence images demonstrated an increase in HL-60 adhesion to endothelial monolayers maintained at 0.0 mM glutamine compared with control cultures (2.0 mM glutamine). (**b**) Quantitative analysis confirmed this observation, showing that glutamine-depleted ECs bound to more than 2.6-fold greater numbers of HL-60 cells relative to controls (*** *p* < 0.001). Groups: 2.0 mM and 0.0 mM; independent experimental replicates N = 4 per group. Scale bar = 700 µm.

We further investigated the effect of glutamine depletion on the promyelocytic–endothelial cell adhesion (Figure 8) which could have potential implication to pathophysiological conditions. Endothelial cells with glutamine depletion showed an increase in HL-60 cell adhesion. Control ECs (2.0 mM glutamine) exhibited adhesion of 5786 HL-60 cells, whereas the glutamine-depleted group (0.0 mM glutamine) showed marked increase in adhesion, with 15,307 HL-60 cells bound to the EC monolayer representing a 2.7-fold increase compared to the control (2.0 mM glutamine). Therefore, glutamine depletion in ECs would lead to increased promyelocytic–endothelial cell adhesion.

### 3.5. Glutamine Depletion Reduced EC Ability to Promote Extra Tissue Growth in Zebrafish Xenograft

**Figure 9:**
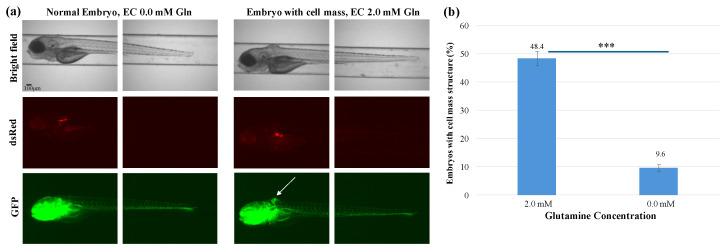
Endothelial cells maintained at physiological glutamine levels promoted increased xenograft cell mass formation in zebrafish embryos. (**a**) Representative images of zebrafish embryos xenografted at the blastula stage with ECs previously maintained in either 0.0 mM or 2.0 mM glutamine conditions. Embryos receiving ECs maintained at 2.0 mM glutamine frequently exhibited localized cell mass formation in the hindbrain region. The white arrow indicates ectopic green zebrafish vasculature associated with the xenograft in the 2.0 mM glutamine group. (**b**) Quantitative analysis of xenografts shown in (**a**) demonstrated more than a five-fold increase in embryos displaying cell mass structures in the 2.0 mM glutamine group compared with the 0.0 mM group, representing a highly significant difference (*** *p* < 0.001). Groups: 2.0 mM (independent experimental replicates N = 3, 84 embryos/group) and 0.0 mM (N = 3, 93 embryos/group). Scale bar = 100 µm.

**Figure 10:**
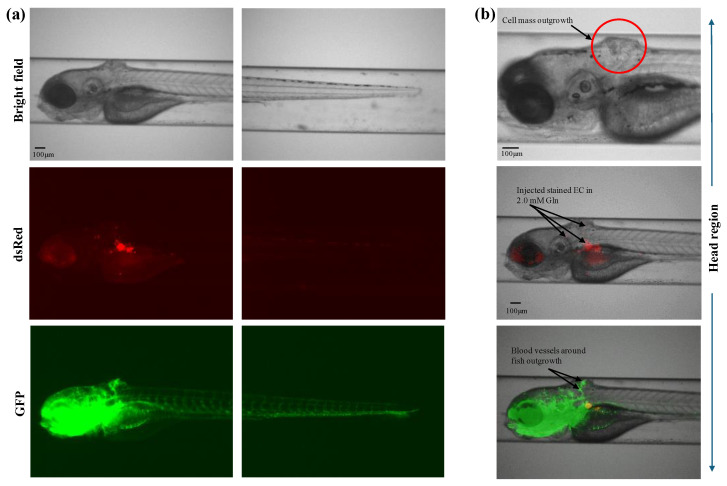
Zebrafish/EC xenografts displaying cell mass formation. (**a**,**b**) Representative images of the same 6 dpf embryo shown in Figure 9a following injection with ECs maintained at 2.0 mM glutamine, visualized in bright-field and fluorescence channels using vertebrate automated screening technology VAST BioImager (Union Biometrica) with Metamorph software (Basic, version 7.8.6.0). (**b**) The bright-field image is shown at two-fold higher magnification relative to the images in (**a**). In the bright-field image (upper panel), the arrow indicates the location of the cell mass. In the dsRed/bright-field overlay (middle panel), arrows indicate CM-DiI-labeled endothelial xenograft cells. In the GFP/dsRed/bright-field composite (lower panel), arrows identify green zebrafish vasculature within the *Tg(fli1:egfp)^y1^* embryo. The head-region overlay in (**b**) shows injected ECs migrating toward the ectopic cell mass and other regions of the embryo. The triple overlay (lower panel) indicates that the mass structure was composed predominantly of zebrafish cells (non-GFP tissue) with possible contributions from migrated human ECs (dsRed-labeled). Green fluorescence within the mass suggests localized zebrafish vascular formation associated with the transplanted ECs (**b**). Scale bar = 100 µm.

Glutamine plays critical and diverse roles by providing not only a source of nitrogen for amino acid and nucleotide biosynthesis but also a source of carbon to replenish the TCA cycle and lipid biosynthesis pathways. Most data presented above were generated from in vitro experiments (Figure 1, Figure 2, Figure 3, Figure 4, Figure 5, Figure 6, Figure 7 and Figure 8). Given that zebrafish share approximately 70% of their genes with humans and possess comparable organs and biological systems, they represent a robust in vivo model to investigate the physiological relevance of glutamine deficiency in EC. Accordingly, a zebrafish xenograft assay was employed (Figure 9 and Figure 10), demonstrating that glutamine-depleted Ea.hy926 ECs had lost their ability to induce host tissue outgrowth accompanied by blood vessel formation. In this study, we examined the result of transplanting glutamine-depleted ECs and control ECs within the zebrafish xenografts. Fertilized eggs at 3 hpf were used for microinjection and at 6 dpf these xenografted embryos were imaged (Figure 9a). Any external outgrowth and cell mass were considered as human EC (red fluorescent) or induced proliferation of zebrafish tissue (green fluorescent in *Tg(fli1:egfp)^y1^* vascular tissue or no fluorescence non-blood vessel) in the xenograft embryo. Results from the xenograft assay demonstrated that control ECs cultured with 2.0 mM glutamine generated cell mass-like growth in more than five-fold as many embryos as glutamine-depleted ECs (0.0 mM glutamine) (Figure 9b), which was in agreement with the statement that glutamine, the most abundant amino acid in plasma, was a well-known nutrient used by rapidly proliferating cells for their growth and survival under metabolic stress conditions [39]. Inducing factors such as the vascular endothelial growth factor could enhance the extra tissue growth, which could be malignant or benign. Human Ea.hy926 ECs grown in 2.0 mM glutamine had the capability to proliferate and migrate inside zebrafish host body. To better illustrate cell mass formation and associated blood vessel development, the same embryo was therefore presented at higher magnification in Figure 10. This analysis showed that the red fluorescent Ea.hy926 ECs and the zebrafish vascular tissues (green fluorescent) were distinguishable and spatially separated when the images were superimposed in Figure 10b, bottom panel. The injected control EC (2.0 mM glutamine) xenograft was shown to have the ability to induce human EC (red fluorescent) as well as zebrafish vascular tissue (green fluorescent) outgrowth in zebrafish (Figure 10a,b). The outgrowth showed that EC morphogenesis had occurred inside, which mainly consists of fish cells and possibly migrated human ECs (Figure 10b). A significantly greater proportion of zebrafish embryos exhibited cell mass-like structures in embryo/EC xenograft in the control group (2.0 mM glutamine), whereas this proportion was reduced to 19.8% in the glutamine-depleted group (0.0 mM glutamine) as compared to the control (Figure 9b). These findings indicated a marked loss of EC morphogenesis and tissue proliferation-inducing capacity under glutamine-depleted conditions. Red fluorescence corresponding to CM-DiI-labeled ECs confirmed the presence of successfully transplanted human ECs in embryo xenografts under both 2.0 mM and 0.0 mM glutamine conditions. However, ECs derived from the 0.0 mM glutamine condition exhibited a marked loss of capacity to induce external outgrowth or cell mass formation. Human ECs were identified by red fluorescence, whereas zebrafish vascular tissues were visualized by green fluorescence *(Tg(fli1:egfp)^y1^)* and non-vascular tissues lacked detectable fluorescence. Notably, induction of EC morphogenesis outgrowth and cell mass formation was observed almost exclusively in embryos xenografted with ECs cultured in 2.0 mM glutamine.

The loss of EC morphogenesis properties in glutamine-depleted ECs was observed in vitro, rendering these cells incapable of tube-like structure formation (Figure 6 and Figure 7). In contrast, control ECs cultured with 2.0 mM glutamine retained their EC morphogenesis potential, exhibiting proper cell migration and lumen-like formation. This distinct vascular morphogenesis pattern was consistent with the findings from our in vivo zebrafish transplantation assay. In control zebrafish/EC xenografts (2.0 mM glutamine), EC morphogenesis and cell mass formation were evident behind the head area. However, these processes were absent in zebrafish/EC xenografts transplanted with glutamine-depleted ECs.

## 4. Discussion

Glutamine is a conditionally essential amino acid that plays a pivotal role in cellular metabolism, proliferation, and survival. Although synthesized endogenously, its demand frequently exceeds supply under physiological stress or pathological conditions, rendering it essential in specific contexts. Importantly, glutamine depletion has been implicated in the pathogenesis of multiple endothelial dysfunction-related diseases [6]. Zhou et al. [6] demonstrated that chronic glutamine depletion activated the Akt-mTOR signaling pathway, suppressing autophagic flux through autolysosome impairment and thereby promoting cellular dysfunction. Likewise, studies in Drosophila have shown that glutamine depletion accelerated phenotypes associated with cellular functional decline. In neurodegenerative diseases such as Alzheimer’s disease, glutamine depletion has been reported in the patient’s brain tissue, while supplementation has been shown to reduce neuroinflammation in Alzheimer’s disease mouse models and extend lifespan in ataxia telangiectasia mutated-deficient mice [40,41]. Collectively, these findings highlight an emerging link between glutamine availability and endothelial dysfunction.

The results presented in Figure 1, Figure 2, Figure 3 and Figure 4 demonstrated a marked increase in SA-β-gal activity in Ea.hy926 EC under glutamine-depleted conditions. Our results on Ea.hy926 ECs were consistent with previous studies demonstrating that cellular dysfunction can be assessed by increased lysosomal SA-β-gal activity through cytochemical staining [42,43]. This enzyme accumulates to a level detectable above background when stained in a mildly acidic solution containing a β-gal substrate at pH 6.0 [42]. The detection of SA-β-gal activity at pH 6.0 is widely used as a marker associated with cellular dysfunction and senescence-like phenotypes. In some contexts, endothelial dysfunction has been associated with phenotypic alterations that include features of the senescence-associated secretory phenotype [4]. The enzymatic activity of SA-β-gal was first identified by Judith Campisi [42], initially observed at pH 4.0 in dermal fibroblasts and epidermal keratinocytes. Campisi also demonstrated that SA-β-gal activity was detectable at pH 6.0 [6]. Furthermore, normal somatic cells undergo an irreversible growth arrest and functional state transition after a finite number of divisions. This mechanism serves as a tumor suppression strategy and is a major contributor to organismal aging [44].

In this study, we demonstrated that even short-term glutamine depletion was sufficient to induce SA-β-gal activity, supported by other defects in EC proliferation, cell morphogenesis, cell adhesion and cell-growth-inducing activity in zebrafish xenografts. Previous studies have shown that glutamine supplementation can attenuate phenotypes induced by oxidative stress [6]. Cellular dysfunction was shown in association with characteristic morphological changes (enlarged, flattened appearance), resistance to apoptosis, chromatin remodeling, and the expression of SA-β-gal [45]. Detection of SA-β-gal activity at pH 6.0 was first reported by Dimri et al. [42]. Human umbilical vein endothelial cell (HUVEC) is a well-established and widely used EC model for investigating vascular biology, cardiovascular disease, and angiogenesis. It has been reported that glutamine depletion could profoundly impair HUVEC function by inhibiting cellular proliferation and migration, as well as disrupting TCA cycle activity. Consistent with our findings, previous studies have reported that glutamine depletion induced an increase in SA-β-gal activity in HUVECs after 7 days of depletion [6]. Our results were consistent with a previous study demonstrating increased SA-β-gal activity in HUVECs after 40 passages [46]. Consistent with these findings, our results revealed a dose- and time-dependent increase in SA-β-gal activity under glutamine-depleted conditions.

Glutamine is indispensable for sustained proliferation and cellular function, whereas its depletion could result in growth arrest and cell death [47]. Although the precise mechanisms remain incompletely defined, glutamine has been shown to regulate cell cycle progression, particularly the G1-to-S phase transition and mitotic entry [48,49]. Our results were consistent with the literature that glutamine can be utilized to provide precursors for purine and pyrimidine nucleotide biosynthesis [1,50], which are essential for energy carriers, nucleic acids and nucleotide cofactors such as nicotinamide adenine dinucleotide and S-adenosylmethionine [51]. Therefore, glutamine can be used as a substrate to support deoxyribonucleic acid synthesis during cell proliferation and cell cycle propagation [52,53]. Glutamine can regulate nitric oxide biosynthesis in ECs [54,55], intact blood vessels [56], and cerebral tissues [57]. Since nitric oxide is an important relaxing factor and signaling molecule in endothelium [58], glutamine was expected to play a critical role in regulating the function of the cardiovascular system [55,59]. Malakar et al. [60] reported that 24–48 h of glutamine depletion significantly reduced proliferation in immortalized human cervical HeLa and breast MDA-MB-231 cancer cell lines. In agreement, our data showed that ECs subjected to glutamine depletion for 5 or 10 days displayed markedly reduced proliferative capacity, even after re-exposure to glutamine-rich (2.0 mM) conditions for 48–96 h (during the proliferation assay).

Beyond its metabolic role, glutamine contributes to cardiovascular health by enhancing endothelial function, promoting nitric oxide synthesis through its role as a precursor for arginine, and exerting antioxidant and anti-inflammatory effects. Glutamine supplementation has been associated with improvements in cardiovascular risk factors, including lipid metabolism, insulin sensitivity, hypertension, and obesity. Conversely, glutamine depletion contributes to endothelial dysfunction and immune dysregulation, both of which are central drivers of cardiovascular disease. Glutamine depletion has been linked to impaired immune responses, as glutamine is crucial fuel for immune cells like lymphocytes and macrophages [1]. Future studies will investigate whether glutamine depletion in immune cells impacts endothelial cell function and contributes to cardiovascular disease. In our Matrigel assays, glutamine-depleted ECs exhibited severe defects in cellular morphogenesis, indicative of compromised angiogenic capacity, thereby supporting the concept that dysfunctional ECs may be particularly vulnerable to cardiovascular pathology.

Emerging evidence also links glutamine depletion to endothelial dysfunction in COVID-19. Clinical studies have shown reduced circulating glutamine levels in COVID-19 patients, correlating with disease severity [36]. This deficit likely impairs immune and endothelial function, contributing to inflammation, coagulopathy, and multi-organ failure. Supporting this, our promyelocytic cell adhesion assays using the HL-60 cell line, a promyelocytic leukemia model capable of differentiating into monocyte-like cells [61], demonstrated more than a 2-fold increase in promyelocytic cell adhesion to EC under glutamine-depleted conditions. These results were consistent with reports that glutamine depletion promoted endothelial inflammation and dysfunction in severe COVID-19 [62,63]. Our findings were consistent with previous evidence showing altered intercellular adhesion molecule-1 expression in late-passage ECs compared with early-passage ECs and in ECs isolated from aged mouse lungs. These findings support the hypothesis that alterations in intercellular adhesion molecule-1 activation and clustering may dysregulate signaling pathways involved in cell–cell interactions and thereby promote immune cell adhesion [64,65].

Angiogenesis is a highly regulated process dependent on endothelial metabolism, extracellular matrix interactions, and growth factor signaling. While glucose metabolism has long been studied in this context, glutamine metabolism has only recently been recognized as critical. Inhibition of glutaminase 1 or glutamine depletion disrupts endothelial proliferation and migration, thereby impairing vessel sprouting [27]. Consistent with previous studies, glutamine consumption was increased in HUVECs and inhibition of glutaminase reduced proliferative capacity and induced SA-β-gal activity in HUVECs [66]. In our zebrafish xenograft model, ECs maintained in glutamine-rich conditions (2.0 mM) displayed robust proliferation, migration, and host tissues outgrowth, including vascular tissues. By contrast, glutamine-depleted ECs failed to induce host tissue outgrowth, reflecting their compromised physiological state.

Our findings are further substantiated by the FDA’s 2017 approval of glutamine supplementation for the treatment of acute complications in sickle cell disease [67]. In sickle cell disease, glutamine supplementation reduces red blood cell adhesion to endothelial cells, thereby improving microvascular function [68,69]. The convergence of evidence across diverse disease models underscores glutamine’s central role in maintaining endothelial integrity and preventing dysfunction.

Glutamine depletion studies using EC lines and zebrafish models have several limitations, including the use of immortalized cell lines, artificial complete glutamine depletion conditions, species-specific differences, and developmental rather than adult physiological contexts. These factors limit direct translational relevance to human vascular biology. Nevertheless, these models provide important insight into the role of glutamine in endothelial cell survival, metabolic homeostasis, and functional integrity, while also improving understanding of pathways linked to cellular deterioration and aging-related vascular dysfunction.

## 5. Conclusions

In conclusion, our study demonstrated that glutamine depletion profoundly impaired Ea.hy926 endothelial cell function, as evidenced by increased SA-β-gal activity, reduced proliferation, enhanced promyelocytic cell adhesion, disrupted morphogenesis, and diminished cell mass formation capacity in zebrafish/EC xenografts. These findings highlight the essential role of glutamine in maintaining endothelial cellular homeostasis and functional integrity. The observed alterations further suggest that glutamine availability is a critical determinant of endothelial function under metabolic stress conditions. Given the close relationships between endothelial dysfunction, immune dysregulation, and metabolic imbalance in vascular pathophysiology, modulation of glutamine metabolism may represent a promising area for future cardiovascular research. Collectively, our data identify glutamine metabolism as an important regulator of endothelial cell function and a potential therapeutic target for preserving vascular health. Further work is required to establish bona fide senescence and define the underlying mechanisms.

## Data Availability

Data is contained within the article. The original contributions presented in this study are included in the article. Further inquiries can be directed to the corresponding author.

## Abbreviations

The following abbreviations are used in this manuscript:

Akt	Protein kinase B
ANOVA	Analysis of variance
CM-DiI	Chloromethylbenzamido-Dioctadecyl-indocarbocyanine iodide
COVID-19	Coronavirus disease of 2019
DAPI	4′,6-diamidino-2-phenylindole
DMEM	Dulbecco’s modified eagle medium
dpf	Day-post-fertilization
Ea.hy926	Immortalized human somatic hybrid endothelial cell
ECs	Endothelial cells
FBS	Fetal bovine serum
HL-60	Immortalized human leukemia-60 cell line
hpf	Hour-post-fertilization
HUVEC	Human umbilical vein endothelial cell
M	Molar
mg	Milligram
ml	Milliliter
mM	Millimolar
mTor	Mechanistic target of rapamycin
μL	MicroliterPBS Phosphate-buffered saline
pH	Potential of hydrogen to specify the acidity or alkalinity
rpm	Revolutions per minute
SA-β-gal	Senescence-associated beta-galactosidase
TCA	Tricarboxylic acid
*Tg(fli1:egfp)^y1^*	Transgenic zebrafish line with fli1 promoter to drive EGFP expression
xg	Times gravity/g-force
